# Implementation of OSCE Test to Develop Appropriate Tool to Measure Mothers’ Parenting Skills

**DOI:** 10.5539/gjhs.v7n2p107

**Published:** 2014-09-28

**Authors:** Saiideh Norouzi, Mohammad H. Baghiani Moghaddam, Mohammad A. Morowwaty Sharifabad, Ali Norouzi, Ali R. Jafari, Hossein Fallah Zadeh

**Affiliations:** 1 Department of Health Education and Promotion, Shahid Sadoughi Yazd University of Medical Sciences, Yazd, Iran; 2 Qazvin University of Medical Sciences, Qazvin, Iran; 3 Department of Psychology, Islamic Azad University Abhar, Iran

**Keywords:** explanatory factor analysis, OSCE, parenting skills, reliability, validity

## Abstract

**Introduction:**

Parents play a vital role throughout a child’s life. This role is very significant in the beginning years of a child’s life. In this period, the child encounters new experiences and these experiences make him or her to learn and grow. These days, in order to help the parents build a bright future for their children, different parenting programs have been designed. These programs provide a great opportunity for parents to enhance positive parenting skills. The aim of this study was to design a reliable and valid instrument for assessing the mothers’ parenting skills.

**Methodology:**

44 mothers who had children aged 3–8 were invited to participate in this study. They took part in parenting-skills training sessions for more than two months. Then they were asked to attend the test center after six months to be tested on trained skills. In this study, mothers’ parenting skills were measured by the OSCE test. The reliability of the test was determined with three methods including split half, Cronbach alpha, and correlation between assessors’ scores in two similar stations. The construct validity of the test was determined with Explanatory Factor Analysis.

**Results:**

The total Cronbach alpha coefficient obtained was 0.83 which indicated that the test had a high internal reliability. The Spearman correlation coefficient obtained for two halves of the test was 0.76. The results of Explanatory Factor Analysis showed that nine stations of OSCE were focused on two factors. The first factor was named positive positions and the second factor was named negative positions.

**Conclusion:**

The designed OSCE test has the suitable psychometric features to be used by researchers to assess mothers’ parenting skills.

## 1. Introduction

Parenting is a complex activity that involves specific methodologies and particular behaviors which influence a child’s growth independently or in interaction with each other (Baumrind, Cowan, & Hetherington, 1991). The parenting program is a positive child management skill set that can be an alternative to inefficient and ineffective parenting practices in training parents (Sanders, Markie-Dadds, & Lynch, 1994).

It is obvious that finding solutions for parents who are under stress due to their child’s education is very important, and therefore the majority of studies designed in this field are parent behavior training interventions (Murrell, Wilson, LaBorde, Drake, & Rogers, 2008). These days, many various interventions in parental behavior trainings are designed. The majority of programs conducted in this field are “behavioral Parent Training”, “Helping the Non-Compliant Child”, “The Incredible Years” and “Positive Parenting Program” (Murrell, 2011).

Helping the Non-Compliant is one of the programs which is proposed by Forehand and McMahon. The target group of this program was mothers with children whose ages ranged between 3-8 years old in which the mothers are educated on the method of correct behavior whilst interacting with their child (McMahon, 2006; McMahon, & Forehand, 2005). This program consists of two phases that are usually implemented in 8-12 meeting-based HNC. Since the HNC program is based on skills, the number of meetings depends on the skill set to be acquired by the parent. In the first phase, there are three skills (Attending, Rewarding, and Ignoring) and in the second phase there are two skills (Giving effective directions, Time-Out).

In the study that was presented by Cann et al in Australia, the participants were 589 parents who took part in a positive parenting program from 1999 to 2003. The results of that study indicated that there was a considerable improvement in efficacy perception of parenting, parental consent and stress anxiety and depression of parental depression. Before the intervention, about 45% of children had behavioral problems that increased to 12% after intervention (Cann, Rogers, & Worley, 2003). The purpose of this study was to construct an appropriate tool for assessing mothers’ parenting skills. This tool is the OSCE test. In this study, we hypothesized that the OSCE test has good validity and reliability in assessing mothers’ parenting skills.

In 1975 Harden and his colleagues introduced the Objective Structured Clinical/Practical Examination (OSCE), claiming that it fulfilled all the criteria of an ideal method of assessment of clinical competence (Barman, 2005). The OSCE test is an objective test which involves approximately four criteria of a fair test (Validity, reliability, objectivity and practicality) (Amin, Chong, & Khoo, 2006; Dent & Harden, 2008). OSCE is one of the most practical tests in the field of evaluating training programs, because it covers all three aspects of cognitive, emotional and mental status. This method of testing has rapid feedback for self - test, it is equal for everybody, and can be an incentive for self-testing and mirror reflection for problems and defects because it is both experimental and practical in a real environment. There is little disagreement that an OSCE style examination provides a more valid assessment of clinical skills than a written or oral examination (Dent & Harden, 2008).

The OSCE has a number of stations in which it is requested from the self-test to do specific skills (Ross et al., 1988). In every station, the individual skills are evaluated by an assessor and a checklist. Chance is in no way implicated in this test, grading is accurate and self-test is able to receive immediate feedback, therefore participants show a greater sense of satisfaction (Ross et al., 1988). The OSCE was first introduced by Harden and Gleeso in 1979 (Dent & Harden, 2008). OSCE was used by the nursery faculty at McMaster University to assess the skills of third-year nursery students in primary care in 1984. The OSCE has been a reliable method in assessing clinical skills for over 30 years (Gorter et al., 2000). It is a means of measuring clinical skills in which the confounding variables can be controlled with it (Carraccio & Englander, 2000).

Many studies within and outside the country have confirmed the reliability and validity of OSCE. In a study conducted by Zargar, for the purpose of examining the validity and reliability of OSCE, it was shown that this method is a reliable and valid one for assessing nursery students’ clinical skills (Zargar, 2004). Also Rkany composed a study on the reliability and validity of functional skills in which the OSCE was introduced as a reliable and valid instrument that provides reliable information about learner performance capabilities (Abdullah et al., 2010). The aim of this study is to assess mothers’ parenting skills by OSCE test for the first time.

## 2. Methodology

### 2.1 Participants

The process of sampling in this study was random sampling. Participants involved 44 mothers with children aged 3 to 8 years. The inclusion criteria were mothers with preschool and early elementary school-aged children, have, at least, diploma literacy and, had completed a written consent form. For ethical reasons, the objectives of this study were explained to the participants and they were assured of data confidentiality. After obtaining completed consent forms, the educational intervention was conducted. All participants took part in nine sessions on parenting skills. The meetings ware weekly and educated mothers on five parenting skills (Attending, Rewarding, Ignoring, Giving effective directions, Time-Out). After a six-month period and the conclusion of meetings, all mothers participated in the OSCE test in order to be tested on their parenting skills.

### 2.2 Measuring Tools

In this study the parenting skills of mothers were measured by the OSCE test. The OSCE test stations were designed by five parenting skills. At first, five skills were divided into positive and negative aspects. Three skills, “Attending, Rewarding, Giving effective directions” that were used in positive situations were referred to as positive subscales and the two skills” Ignoring, Time-Out” that were used in negative situations were referred to as negative subscales. The next step was to design an OSCE test station which consisted of 9 stations, 5 stations of which were measured on positive situations and 4 on negative situations. Each station is made up of four components (Scenario, trained raters, trained children and check list). It should be mentioned that the Attending skill was measured in one station only because of the lack of assessors and facilities.

### 2.3 Scenarios

Considering the five principles of parenting skills, 9 operating scenarios were developed for OCSE stations. Five scenarios were written according to positive aspects and four seniors according to negative aspects. This scenario determined what behavior was expected from trained children when their mothers enter the OSCE station. The following is an example of the positive and negative aspect of scenarios.

#### 2.3.1 Negative Aspects of Scenarios (Time-Out 1)

Suppose that the child who is in the room is your child. He constantly uses inappropriate language and exhibits malevolent behavior. You have repeatedly asked him not to do so, but despite this he has continued. You have decided to punish him. Please punish him using Time-Out skill.

#### 2.3.2 Positive Aspects of Scenarios (Attending)

Suppose that the child who is in the room is your child and during the last week you have several times heard from him;” You do not pay any attention to me” so you decide to show that this is a wrong belief and you have paid enough attention to him. He is currently drawing. Please implement the Attending skill on him.

### 2.4 Assessor

Nine specialists in the field of educational psychology were selected as the assessors of this test. Firstly, the purpose of the study was fully explained to them, then the implementation of the OSCE test was explained. Finally, they began doing the test with trained children. Then the assessor of every station had to evaluate the situation of the mother after entering the station. Then the assessor asked the mother to envision that the child who is in the room is her child. Next, according to the behavior of the child, she performs good parenting skills on him or her. When the mother is performing the skills, the assessor will then evaluate by the check list.

### 2.5 Trained Children

Nine imaginary children aged 3 to 8 were selected in the OSCE stations. There was a specific scenario selected for every child and its procedures were fully explained. The children practiced all the scenarios for several sessions along with their respective assessors.

### 2.6 Checklists

There was a checklist in each station for evaluating the parenting skills of mothers. The questions of the checklists were YES and NO questions that were evaluated by the assessors after observing the mothers’ skills while dealing with the child. The list below is a sample of the checklist:

### 2.7 Procedure

Having provided the essential facilities for administering the OSCE test and having held some training sessions with trained children and assessors, the test was administered. The trained mothers were required to arrive at a certain time at the test center.

Once the mothers arrived at the place, they entered the OSCE stations and their skills were assessed with the checklist by the assessors. 9 checklists were completed for each mother.

In the next phase, the checklist was scored. Each YES answer was given 1 point while the NO answer was given 0 point. The scores of each station were separately collected and consequently each mother obtained 9 different scores from 9 stations.

## 3. Results

The data obtained from the study was analyzed by the SPSS software.

### 3.1 Reliability

One of the problems that have always threatened the OSCE test has been the effects of difference the assessors create. If not handled appropriately, it can have irreparable effects on reliability of the test. In the present study, in order to determine the reliability of the scores between assessors, each skill was evaluated by two assessors in two separate stations and with two different scenarios. Hence, the inter-rater reliability was to be consolidated if there was a correlation between obtained scores from two similar stations.

The split method was used to determine the reliability of the test. Given that each skill was examined in two separate stations, the first half consisted of the first stations and the second station was in the second half. The Spearman test showed that the correlation between the two halves was (0.76). To calculate the reliability of the scores obtained from the positive and negative aspects of the test, the Cronbach’s alpha coefficient was used. The Cronbach’s Alpha coefficients obtained for the positive and negative aspects of the test are shown in Table 4.

**Table 1:** Distribution of skills in OSCE Stations

Stations	Positive/Negative Aspect	skills
1	Positive	Rewarding 1
2	Negative	Ignoring 1
3	Positive	Giving effective directions 1
4	Negative	Time-Out 1
5	Positive	Rewarding 2
6	Negative	Time-Out 2
7	Positive	Giving effective directions 2
8	Negative	Ignoring 2
9	Positive	Attending

**Table 2:** Checklist for attending skill

Row	Instructions	yes	no
1	She sits next to the child		
2	She precisely reports the behavior of the child or imitates the child		
3	She does not command when she is attending to him		
4	She does not prohibit the child from anything when she is attending to him.		
5	She does not ask him any questions when she is attending to him.		

**Table 3:** The correlation between the scores obtained from two similar stations

Two similar station	The correlation between the scores of two same station	
Rewarding 1,2	r=0.49	P=0.001
Effectively give directions1,2	r=0.64	P=0.0001
Ignoring1,2	r=0.85	P=0.0001
Time-Out 1,2	r=0.92	P=0.0001

**Table 4:** Coefficient alpha obtained from the positive and negative aspects of the situation

	Cronbach’s Alpha	Items
Positive aspects	0.77	5
Negative aspects	0.94	4
Total	0.83	9

### 3.2 Validity

The Exploratory factor analysis method was used to examine the construct validity of the test. For this reason, the applicability of factor analysis of the data was examined. The results of the Bartlett test and sample adequacy (KMO) in Table 5 shows that Factor analysis is a characteristic feature.

**Table 5:** The results of the Bartlett test and sample adequacy and (KMO)

Kaiser-Meyer-Olkin Measure of Sampling Adequacy.	0.719
Bartlett’s Test of Sphericity Approx. Chi-Square	278.552
Df	36
P(sig)	0.0001

**Table 6:** Results of exploratory factor analysis

Stations	Factors	
	1	2
	
Time-Out,2	0.944	
Ignoring,2	0.943	
Time-Out,1	0.907	
Ignoring,1	0.888	
Rewarding,1		0.802
Effectively Give Directions,1		0.700
Effectively Give Directions,2		0.698
Attending,1		0.698
Rewarding,2		0.665

In order to undertake a factor analysis, principal component analysis and Varimax rotation method were used. Two factors were obtained with Eigen values greater than one which explained 70.59% the total variance of the variables.

**Figure 1:**
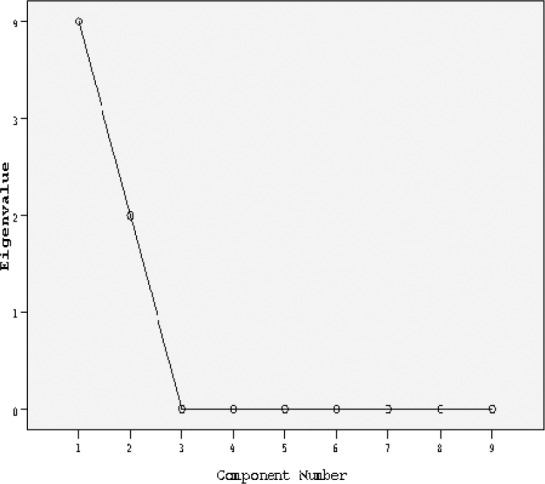
Scree plot indicated that the two factors are responsible for most of the total variance explained

Table 4 shows the results of exploratory factor analysis. As can be seen, 9 subscales has focused on two factors and has formed the two halves of the test. The first factor included stations 2, 4, 6 and 8, which indicate negative positions of parenting skills. The second factor included stations 1, 3, 5, 7 and 9, which indicate positive positions of parenting skills.

## 4. Discussion

Parenting is a complex activity that involves specific methods and behaviors that affect a child’s development separately or in interaction with each other (Baumrind et al., 1991). The aim of parenting education is to assist the parent (usually the mother) for an appropriate response in different situations (Sanders et al., 1994). Researchers in the field of family and school education have attempted to understand the relationship between the methods of parenting and academic performance and to show how parenting methods influence academic performance in their study (RSanders & Morawska, 2007). Recent research shows that parenting is not necessarily a natural skill and most parents need parenting skills training (Dornbusch, Ritter, Leiderman, Roberts, & Fraleigh, 1987). The motive for the construction of this study is to assemble an appropriate instrument to measure maternal parenting skills using the OSCE so as to be able to properly evaluate the skills that are acquired by mothers after parenting education programs. In 1990, Miller provided a framework in which the levels of a pyramid were indicated by four levels of clinical skills (Knows, Knows How, Shows and Does). Accordingly, if we want to evaluate how to perform a skill by learning, it is essential to use the tools at the levels of Shows and Does. In this Pyramid the OSCE tool is located in the level of shows (Amin et al., 2006).

Considering that the aim of this study was to assess mothers’ parenting skills, it was decided that the tools available at the top of the Miller pyramid should be used for the first-time to measure the mothers’ parenting skills used in the OSCE test.

A large number of studies have examined the reliability of the OSCE test. Various researchers have used a variety of methods to estimate these factors. Nikbakht et-al attempted to determine the reliability using the Spearman’s test in their study. In that study, each station was independently administered by two raters who each had a list of checklists of participants to score their performance (Nickbakht, Amiri, & Latifi, 2013). Rozena and Graham used Cronbach’s alpha to determine reliability in their study. In that study, different methods were used to obtain the reliability of the OSCE test all of which indicated that the reliability of OSCE was good (Graham, 2010). To eliminate the difference between the scoring evaluations of each skill on two separate stations, every skill was measured in two separate stations with two different scenarios. It was expected that if the reliability of the scoring was good, participants would receive similar scores for the same station. The results showed that another method was used to determine reliability, which was the split-half method. The results of the Spearman coefficient showed that there was a high correlation between the two halves. Rkani’s study determined predictive and concurrent validity. To determine the predictive validity, he compared the final score of the OSCE test with the results that were achieved from the special skills of each participant 2 months after the OSCE. To determine concurrent validity, he compared the score at the end of each station with the overall performance score at end of every station (Abdollah J. Rekany). The content validity of Rozena and Graham’s study were appointed by three experienced clinical faculty members.

The predictive validity and cohesion was determined by correlation participants’ scores in OSCE and their performance scores during the next 3 years (Graham, 2010). The mothers’ scores at the same station were highly correlated with each other.

In this study, in order to determine the construct validity of the measurement instrument, exploratory factor analysis was used. According to the study, the mother’s parenting skills were assessed at 9 stations and so in the end, 9 grades were obtained.

The resulting scores were entered into SPSS software and were analyzed to determine the validity. Nine OSCE stations focused on two factors, and these two factors accounted for 70.59% of the total variance in the data. The first factor included stations 1, 3, 5, 7 and 9, which fills all the stations indicating positive positions of parenting skills. The second factor included stations 2, 4, 6 and 8, which fill all the stations indicating negative positions of parenting skills.

## 5. Limitations

The most obvious point about this research was designing a suitable instrument for assessing mothers’ parenting skills by means of the OSCE test in Iran. Despite this, it had some delimitations which are mentioned below: The lack of assessors caused the lack of OSCE stations. The preparation and designing process took a long time. Also the training process of assessors took a long time and cost a huge sum of money.
